# Modelling the significance of food delivery service quality on customer satisfaction and reuse intention

**DOI:** 10.1371/journal.pone.0293914

**Published:** 2024-02-15

**Authors:** Mengling Wu, Jingzu Gao, Naeem Hayat, Siyu Long, Qing Yang, Abdullah Al Mamun

**Affiliations:** 1 UKM—Graduate School of Business, Universiti Kebangsaan Malaysia, Selangor, Darul Ehsan, Malaysia; 2 Global Entrepreneurship Research and Innovation Centre, Universiti Malaysia Kelantan, Pengkalan Chepa, Kelantan, Malaysia; HUTECH University, VIET NAM

## Abstract

The millions-worth revenue derived from large-scale food delivery characterises the service as a relatively established phenomenon with potential growth. The current cross-sectional research examined online food delivery service quality on consumer satisfaction and reuse intention. Service quality was divided into seven categories (i.e., reliability, assurance, security, maintaining food quality, system operation, traceability, and perceived service value). Perceived service value offer the unique understanding of the online food delivery consumer satisfaction. Empirical data were elicited from 1352 valid respondents and subsequently assessed through the partial least square structural equation modelling. Findings revealed that reliability, assurance, maintaining food quality, system operation, traceability, and perceived service value could elevate customer satisfaction and optimize the intention to reuse food delivery services. Specific measures to improve service quality, including staff training, improved after-sales service, and system optimisation, were proposed to increase users’ satisfaction and intention to reuse optimally.

## Introduction

The drastic shift in individuals’ food, clothing, housing and transportation habits has proved advantageous given the rapid growth of the Internet and technologies involving big data, such as online food delivery services [1]. Such extensive data-driven services cater to local lifestyles based on consumers’ needs to establish online and offline consumption scenarios, achieve a closed loop of online transactions, and fulfil offline transactions through instant delivery [2]. In this vein, customers are offered a one-stop service from demand initiation to goods acceptance. A substantial number of restaurants are currently providing food delivery services through brand-developed programmes or digital food ordering platforms (Meituan and Foodpanda) with the development of e-commerce [3]. The global online food delivery market, which was worth US$82 billion in 2018, has increased by up to 140% following the advent of COVID-19 and the subsequent imposition of multiple anti-epidemic policies and travel restrictions [4].

Online food delivery (OFD) utilises the Internet and digital information platforms as mediums to connect consumers with offline catering enterprises. The integration of this user demand-oriented service with takeout resource integration provides consumers with much food-ordering information and convenient food delivery services [5]. In expanding this novel business model, catering enterprises could offer customers unique sales and marketing channels through online ordering and digital food delivery services [6]. The emergence of online food delivery platforms that enable food orders beyond time and space inevitably transforms conventional dining. It renders convenient for consumers to fulfil their basic needs amidst hectic schedules [7]. The number of online food delivery users in China reached 521 million in December 2022 (an increase of 102 million from December 2020), accounting for 48.8% of all internet users [8]. Overall, the continued expansion of online food delivery market size, dynamic and competitive landscapes, and technological innovation application enhancements in delivery catalysed online takeaway platform development. However, it requires more understanding towards formation of online food delivery consumer satisfaction with the factors of online food delivery services that may leads to reuse of the online food delivery services.

The service quality impact on the competitiveness of the online food delivery sector proves significant as this industry primarily offers consumers meal delivery services [4]. Online food delivery service quality has gained prominence following the multitude of digital food delivery platforms to choose from and rapid industrial growth [7]. Hence, the relatively disregarded service quality in the past is an essential factor impacting customer satisfaction (SAT) and intention to reuse (ITR) OFD apps (also referred to as applications) [9]. The key determinants of online food delivery service quality should be considered to elevate customer SAT while establishing a competitive organisational edge to achieve a win-win situation for both users and companies.

Past research emphasised the factors influencing customers’ SAT and repeated use of takeaway platforms [10–12]. Online service providers must provide a seamless delivery of all phases, from searching and order placement to payment and delivery, given the complexities underpinning digital food delivery with multiple steps of contact [5]. Cheng et al.’s [4] review of past service quality scales categorised takeaway service quality into six dimensions, including reliability (REL), maintaining food quality (MFQ), assurance (ASE), security (SCT), system operation (SOP) and traceability (TRY); the scale failed to assess service quality from the perspective of customers’ perceived value. The perceived value (PSV) level would directly impact customers’ (service recipients) SAT towards service quality. Despite much research on the PSV-SAT link, PSV has been relatively disregarded in service quality-oriented works [6]. As such, this study utilised PSV as one of the dimensions to measure takeaway service quality, which further examined the customer service quality implications on their SAT and ITR in the takeaway industry. The extent to which (i) REL, ASE, SCT, MFQ, SOP, TRY, and PSV affected customer SAT in using takeaway platforms and whether (ii) such SAT affected consumers’ ITR were also investigated in this study, and the mediational effect of SAT between the seven dimensions of service quality and consumer ITR. The current work, which focused on a nation with well-established digital and takeout industries, i.e., China, assessed this user group for insights into the critical determinants of consumers’ ITR of takeout sites.

## Literature review

### Theoretical foundation

Parasuraman et al.’s [13] SERVQUAL model, which divides service quality into five dimensions (REL, ASE, tangibility, empathy, and responsiveness), is one of the most extensively employed product or service quality assessment methods. SERVQUAL indicates more optimal service performance than customer expectations regarding the gap between clients’ service-oriented anticipations and impressions [13]. The five service quality dimensions are defined as follows: (i) REL denotes performance consistency and dependability; (ii) ASE represents employees’ expertise, courtesy and their ability to establish client trust and confidence; (iii) tangibility constitutes service providers’ physical facilities, equipment, and appearance; (iv) empathy characterises the personalised customer attention and care; (v) responsiveness implies service providers’ eagerness or preparedness to perform prompt service delivery. Parallel to past works, the SERVQUAL model is associated with or influenced customer SAT in multiple sectors [2, 14]. In this vein, service quality plays a crucial role in the organisational competition, given the drastic rise of digital delivery platforms. The online food delivery industry attributes entail system operation, company delivery and the delivery service provided by the delivery man [1]. Consumers’ PSV should also be regarded as a relevant service quality factor and core component of service generation [6]. By integrating the SERVQUAL model with the online food delivery industry attributes, this research proposed seven dimensions: REL, ASE, SCT, MFQ, SOP, TRY, and PSV to assess service quality and examine its effect on customer SAT.

### Hypotheses development

#### Reliability and satisfaction

As the consistency of the final service performance and service provider commitment [13], REL in the online food delivery context denotes the deliveryman’s efficient completion of the pre-determined delivery service, such as ensuring that the goods are in good condition, the bill is accurate, and the service attitude is positive [15]. A deliveryman’s service quality, which links the delivery platform and the customer, is a crucial element that directly impacts users’ perceived quality. It is deemed convenient for delivery service platforms to communicate with consumers, earn their client’s trust, and elevate users’ SAT when the deliveryman offers high-quality and professional customer service. Likewise, Cheng et al. [4] ascertained REL as a key determinant of food delivery service quality. Past literature also demonstrated that customers who perceive higher REL with food delivery providers tend to experience high SAT levels and make repeat purchases [15, 16]. In this regard, the following hypothesis was developed:

*H*_*1*_: *The REL positively influences customer SAT*.

#### Assurance and satisfaction

ASE implies customers’ confidence in utilising online platforms owing to its reputation, the items or services on sale, and clear and genuine information [17]. As the embodiment of product and service values in e-commerce and technology domains, ASE [18], in the context of online food delivery platforms, denote the degree to which a food delivery platform earns customer trust in service delivery. When a user orders from online food delivery applications, guarantees of accuracy, delivery time, and reasonable delivery fee potentially impact users’ trust in the food delivery platform and perceived ASE [9]. Generally, prompt food delivery and reasonable delivery costs serve to enhance customers’ ASE. This study proposed the following hypothesis based on Kian et al.’s [15] identification of a positive ASE-SAT relationship:

*H*_*2*_: *The ASE positively influences customer SAT*.

#### Security and satisfaction

The SCT denotes consumers’ sense of feeling when shopping online and cognizance that their personal information is safe from third parties [19]. Customers’ concerns about personal data leakage when using electronic platforms, specifically regarding financial issues, is one of the primary reasons deterring them from using electronic platforms amidst rapid, significant data developments [20]. Regarding business-to-customer connections, consumers highly prioritise online purchase SCT in accepting transaction-related risks [21]. To ensure privacy, individuals must frequently fill in accurate and effective delivery addresses, personal contact information, and payment information when using food delivery platforms. Data leakage could instigate loss of customer property or even personal safety. Empirically, users’ tendency to use e-service systems or applications was positively influenced by perceived security [20]. The following hypothesis was developed following Kian et al.’s [15] assertion of SCT as the key determinant of customer SAT:

*H*_*3*_: *The SCT positively influences customer SAT*.

#### Maintaining food quality and satisfaction

The food delivery platform’s ability to guarantee high-quality food that is unaffected by the delivery service process, such as transportation, implies MFQ [16]. Given its inevitability in the food delivery process, it is crucial to determine strategies to maintain and ensure high food quality during delivery. Prolonged delivery time and damaged packaging potentially impact the food taste. Notably, the waiting time and trial-and-error costs for online food delivery are longer and higher than for dine-in services. Consumers who complain about food delivery issues would experience low SAT levels. Following Kian et al. [15], MFQ is crucial to customers’ SAT with online food delivery services. Likewise, Al Amin et al.’s [22] study implied that consumers’ concerns about the delivered food quality would impact their product SAT and ITR of the food delivery platform. As users are prone to reuse such digital sites with high trust levels in the food quality during the delivery process, the following hypothesis was developed:

*H*_*4*_: *The MFQ positively influences customer SAT*.

#### System operation and satisfaction

Effective technical application operations or SOP [17] in the food delivery platform context involves search, reservation, order placement, delivery, and after-sales. Notably, SOP-oriented issues would inevitably increase the process complexities. Seamless system operations enable customers to efficiently and quickly identify desired meals and place orders, elevating customer SAT. Strzelecki and Rizun [19] indicated that seamless and convenient application systems could provide users with a better experience, such as rapid screening, product and service comparison, and suitable product or service selection to increase loyalty and trust towards the application and propensity to reuse the platform. This study proposed the following hypothesis as visual and system design and information quality significantly affect consumers’ attitudes towards online food delivery services [23, 24] following relevant research:

*H*_*5*_: *The SOP positively influences customer SAT*.

#### Traceability and satisfaction

Essentially, TRY denotes the technological capacity to track and enquire about delivery progress for online food delivery [11]. TRY allows users to monitor the order delivery status in real-time, ensure smooth food delivery, protect user interests, maintain a good customer experience, and elevate their SAT [25]. For example, the TRY system enables consumers to receive information from the deliveryman through a communication platform to mitigate their anxiousness and waiting time. In this regard, TRY is one of the most pivotal factors influencing customers’ SAT with online delivery platforms [4, 15]. Cheng et al. [4] revealed TRY as one of the indicators for users to evaluate online delivery service quality. The study also demonstrated a significant and positive influence on customer SAT. In line with Choi [11], TRY’s online food delivery system potentially enhances users’ shopping experience by rendering it more productive, enjoyable, and satisfying. Thus, the following hypothesis was developed:

*H*_*6*_: *The TRY positively influences customer SAT*.

#### Perceived service value and satisfaction

A customer’s overall estimation of service utility based on perceptions of what is received and supplied denotes PSV [26]. Specifically, PSV in the online food delivery context implies users’ expected benefits from the platform services. As cost and value establish customers’ perception of service value, a high PSV could encourage positive consumer attitudes and high SAT levels. Bonsón Ponte et al. [27] opined that a positive online service experience could create value for users and improve customers’ SAT. This study proposed the following hypothesis in line with Aslam et al. [6], who proved that high PSV rendered it easier for online food delivery applications to impact customers’ SAT and ITR positively:

*H*_*7*_: *The PSV positively influences customer SAT*.

#### Satisfaction and intention to reuse

User SAT defines consumers’ emotional state following an overall (i) product or service assessment or (ii) a cognitive assessment of perceived quality and emotional attributes induced by their consumption experiences [28]. Users would be pleased with their experience if the actual outcomes of employing food delivery service mirror or surpass their expectations. Consumers who enjoy their food delivery service experience are inclined to continue using such apps. Oliver [29] highlighted customer SAT as a primary ITR predictor. Meanwhile, Alalwan’s [10] research involving the e-SAT impacts on continued ITR in mobile food ordering apps disclosed that improved customers’ SAT promotes their ITR of food delivery services. In this vein, this study proposed the following hypothesis:

*H*_*8*_: *Customer SAT positively influences the ITR of online food delivery services*.

#### Mediational effect of satisfaction

Effect and consistent delivery of the services and product promote the perception of reliability for the product or services [1]. The perception of reliability among customers can harness customer satisfaction that leads to repeat purchases [4]. Assurance comes when the customer feels confident using the product and services in the e-commerce platform. Annaraud and Berezina [9] postulated that the assurance of delivery services builds the e-services users’ satisfaction. The positive assurance leads to satisfaction that can promote the reuse intention. E-services are commonly associated with customer security concerns, as customer financial and personal information data may come at risk [20]. However, the appropriate level of security measures can lead to the positive emotion of customer satisfaction. The perceived security also can enhance the reuse intention through the satisfaction achieved by the security measurements. Maintenance of food quality becomes a salient feature of food delivery services that promotes customer satisfaction [22]. However, estimating the mediating effect of satisfaction between maintaining food quality and the reuse intention for food delivery services is necessary.

Moreover, the food delivery systems require an online delivery application that empowers the users to search, place orders and receive food delivery at specified locations [24]. System operations are a necessary part of food delivery services. The operation of the food delivery system facilitates customer satisfaction, which can promote the reuse intention among food delivery users. Another significant aspect of food delivery services is the traceability of the delivery man. The food delivery customer rated the traceability of the food delivery highly and considered a food delivery services dimension that elevates customer satisfaction. However, estimating the mediating effect of customer satisfaction in the relationship between traceability and reuse intention for food delivery services is interesting. Lastly, the perception of service value positively influences customer satisfaction. A positive service experience can nurture elevated customer satisfaction that can promote the reuse intention of the food delivery services.

Therefore, the following mediational hypotheses are proposed.

H_M1-7_: The relationship between the REL, ASE, SCT, MFQ, SOP, TRY and REL with ITR mediated by SAT.

**Fig 1:**
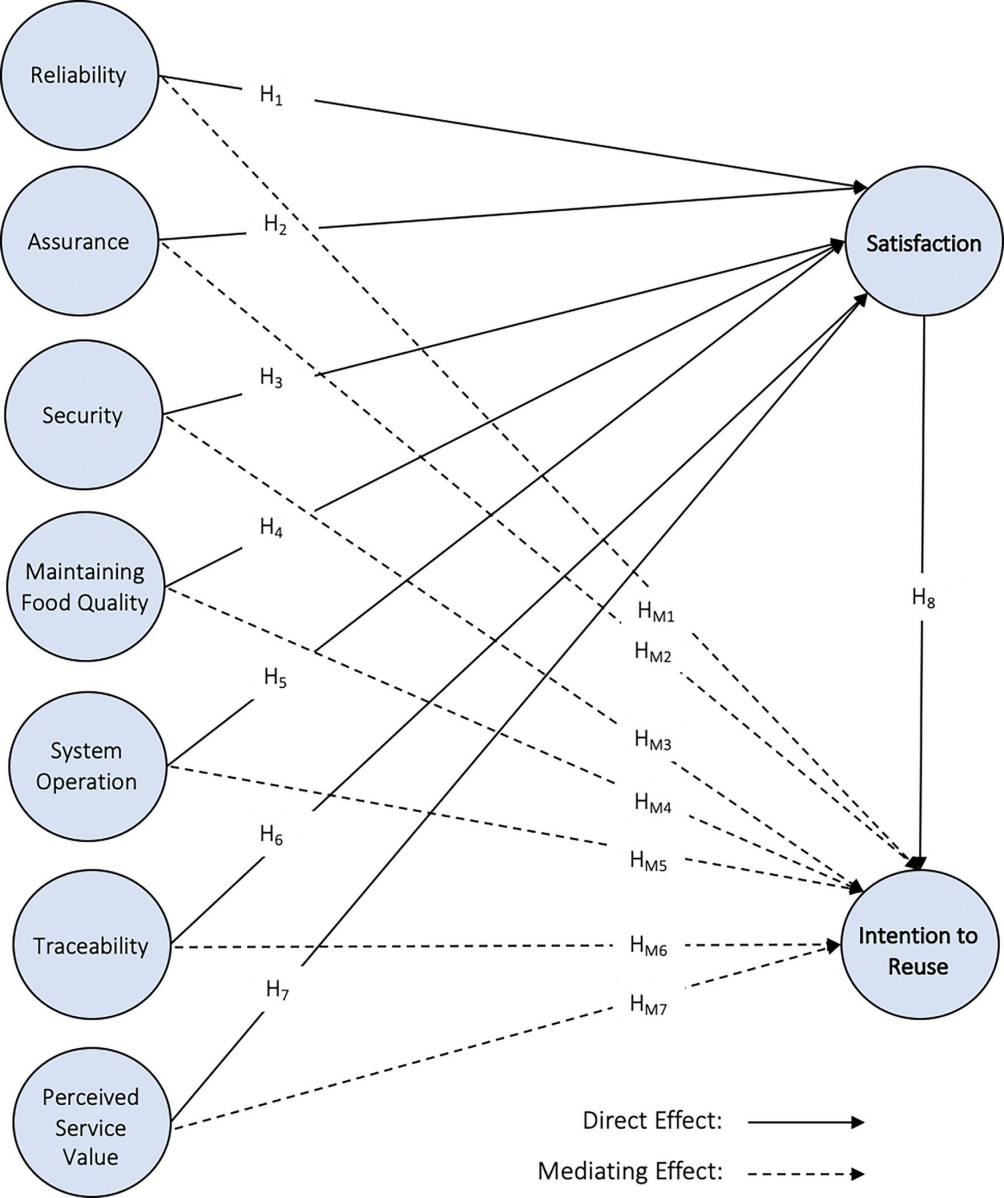


All associations hypothesised above are presented in Fig 1 below:

## Research methodology

This quantitative cross-sectional study employed the questionnaire survey method, which was disseminated to individuals with experience using food delivery applications in China (target group). The study data were gathered from March to June 2022 using an online questionnaire collection platform (Questionnaire Star). A non-probability judgment sampling technique was chosen for scholars to select suitable respondents better. The respondent needs to answer the qualifying question that they use the food delivery application. Respondents were also required to accept the consent form to take a voluntary part in the current study. Additionally, Smart PLS modelling was incorporated for variable correlation analysis. Empirically, the sample size should not be under 200 when utilising PLS-SEM for data assessment to ensure optimal model development [30]. A total of 1325 valid questionnaires were eventually chosen for relevant data analysis upon screening the digitally-derived outcomes.

### Survey instrument

The current study tool is divided into two sections: (i) demographic details (gender, age, education level, marital status, location, occupation, monthly income level, and sample screening questions) and (ii) variable measurement with a seven-point Likert scale ranging between ‘strongly disagree’ to ‘strongly agree’. All the research variables are presented as follows: REL, ASE, SCT, MFQ, SOP, TRY, PSV, SAT, and ITR. Notably, the original version of the study questionnaire, translated from English to Chinese by a professional translation agency, was subjected to a pre-test. The study items were adapted based on current research questions. Specifically, the items involved in REL, ASE, MFQ, SOP, and TRY were adapted from Cheng et al. [4], those of SCT from Cheng et al. [4] and Kumar et al. [31], PSV from Kim et al. [32], SAT from Cappelli et al., [33] and Choi [11], and ITR from Kim et al. [32] and Kang and Namkung [34]. S1 Table. Survey Instrument presents all the current study items.

### Common Method Bias (CMB)

The test outcomes between structures may be biased owing to CMB when using questionnaires for data collection. Harman’s single factor is a post-hoc approach that identifies possible CMB issues [35]. The outcome derived from Harman’s single factor (45.001%) proved lower than the proposed threshold of 50%. Kock’s [36] full collinearity assessment approach was also performed in this work. Following Table 1, the variance inflation factor (VIF) value of under 3.3 in this study implied no CMB.

**Table 1:** Full collinearity test.

Variables	REL	ASE	SCT	MFQ	SOP	TRY	PSV	SAT	ITR
VIF	1.756	1.559	1.585	1.641	1.605	1.542	1.556	1.509	2.229

**Note:** REL: Reliability; ASE: Assurance; SCT: Security; MFQ: Maintaining Food Quality; SOP: System Operation; TRY: Traceability; PSV: Perceived Service Value; SAT: Satisfaction; ITR: Intention to Reuse.

### Multivariate normality

Multivariate normality issue was assessed in this study pre-statistical analysis in line with Hair et al. [30]. The *p*-value of Mardia’s multivariate skewness kurtosis, below 0.05, denoted data non-normality and complemented the utilisation of SmartPLS for data evaluation.

### Data analysis method

This study evaluated data using multivariate analysis, specifically structural equation modelling (SEM), a variance-based partial least squares SEM (PLS-SEM) with maximum likelihood estimation. As a means of examining the links between abstract notions that function with intricate constructions and higher degrees of abstraction, PLS-SEM generates higher construct reliability and validity that complement composite-based models, the model reliabilities were estimated with the Cronbach Alpha, composite realibility and DG rho, convergent validity was estimated average value extracted (AVE), discriminant validty postulated with the Fornell-Larcker criterion, Hetro-trait Mono-trait ratio and cross-loading [30]. The model performance criteria utilized in the study were *R*^2^, Q^2^ and the effect size (*f*^2^), and the path analysis was estimated with the path value, t score and significance level [30].

This study utilized the transmittal strategy to estimate the mediational relationships [37]. Hair et al. [38], guidelines was employed to evaluate the mediation analysis, where the bootstrapping was performed and the confidence level for the path must be assessed. If zero doesnot come between the lower and higher values of confidence level. It suggest the presence of mediation effect of a mediator between the input and comome variable [37].

## Result

### Demographic characteristics

The demographic attributes of all 1352 respondents are presented in Table 2. Gender-wise, 705 respondents (53, 2%) are male, while the remaining 620 (46.8%) are female. In terms of age group, 367 (27.7%) respondents were under 20 years old, 484 (36.5%) were from 20 to 30 years old, 234 (17.7%) were between 31 and 40 years old, 160 (12.1%) were from 41 to 50 years old, and 80 (6%) were 51 years old and above. Regarding marital status, slightly over half of the respondents were married (51.3%), 584 (41.4%) were single, and the remaining few were either divorced (6.0%) or widowed (1.3%).

**Table 2:** Demographic profile of respondents.

	n	%		n	%
*Gender*			*Average Monthly Income*		
Male	705	53.2	Less than RMB1500	277	20.9
Female	620	46.8	RMB1500-RMB3000	464	35.0
Total	1352	100.0	RMB3001-RMB4500	193	14.6
			RMB4501-RMB6000	167	12.6
*Age Group*			RMB6001-RMB7500	96	7.2
Below 20 years old	367	27.7	More than RMB 7500	128	9.7
20–30 years old	484	36.5	Total	1325	100
31–40 years old	234	17.7			
41–50 years old	160	12.1	*Region*		
51 years old and above	80	6.0	Northeast China	147	11.1
Total	1325	100	North China	212	16.0
			East China	283	21.4
*Marital Status*			Central China	160	12.1
Single	584	41.4	South China	229	17.3
Married	680	51.3	Northwest China	150	11.3
divorced	80	6.0	Southwest China	112	8.5
Widowed	17	1.3	Others	32	2.4
Total	1325	100	Total	1325	100
*Occupation*			*Monthly Usage of Food Delivery Application*		
Unemployed	33	2.5	Rarely or not use	109	8.2
Self-employed	173	13.1	1 to 5 times	590	44.5
Student	633	47.8	6 to 10 times	373	28.2
Housewife	46	3.5	11 to 15 times	180	13.6
Privately employed	349	26.3	More than 15 times	73	5.5
Public servant	91	6.9	Total	1325	100
Total	1325	100			
*Education Level*					
College degree and below	374	28.2			
Bachelor or equivalent	605	45.7			
Master or equivalent	288	21.7			
PhD/DBA	58	4.4			
Total	1325	100			

**Note:** 1 USD = 7.24 RMB

With regards to occupation, 33 (2.5%) of the individuals were unemployed, 173 were self-employed (13.1%), 633 were students (47.8%), 46 were housewives (3.5%), 349 were privately employed (26.3%), and 91 were government staff (6.9%). Concerning education level, a significant number of respondents held a Bachelor’s degree (45.7%), 374 (28.2%) were college graduates and below, 288 (21.7%) held a Master’s degree, and 58 (4.4%) were PhD holders or held other qualifications. Based on average monthly income, 277 (20.9) of the respondents earned under RMB1500, 464 (35%) earned from RMB 1500 to RMB 3000, 193 (14.6%) earned from RMB 3001 to RMB 4500, 167 (12.6%) earned from RMB 4501 to RMB 6000, 96 (7.2%) earned from RMB 6001 to 7500, and 128 (9.7%) earned over RMB 7500. Region-wise, most respondents hailed from East China (283, 21.4%), while other counterparts originated from different regions in China. Regarding monthly usage, 109 (8.2%) of the individuals rarely or have never used food applications before, a substantial number used them between 1 to 5 times, 373 (28.2%) between 6 to 10 times, 180 (13.6%) between 11 to 15 times, and 73 (5.5%) over 15 times.

### Validity and reliability

**Fig 2:**
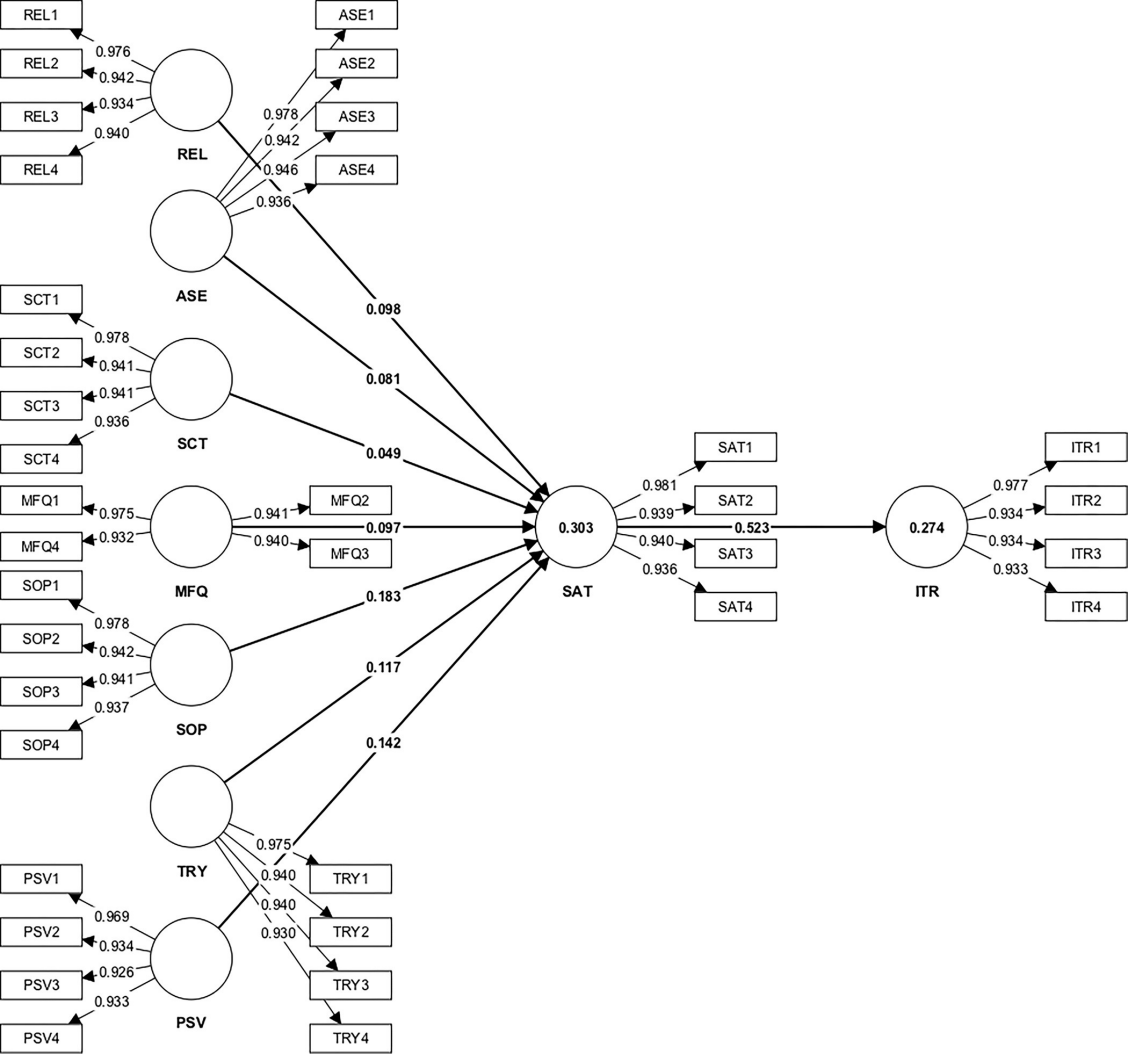


This research validated the internal and external consistency of study constructs through Cronbach’s alpha, Dijkstra-Hensele’s *rho_A*, and composite reliability with a threshold value 0.7. Following Table 3, each construct measurement in this study exceeded 0.7 and proved internally consistent. Meanwhile, the average variance extracted (AVE), which exceeded 0.5, implied convergent validity. The HTMT in this study varied between 0.360 and 0.566 (see Table 4), which was below the threshold value of 0.85. All the square roots of AVEs were greater than the correlation coefficients among the constructs, thus implying discriminant validity [39]. *S2 Table Loading and Cross Loadings* and Fig 2 present all the loading values, which exceeded 0.7 and surpassed the cross-loading values. Resultantly, the discriminant validity of all the study items was confirmed [30].

**Table 3:** Reliability and validity.

Variables	Items	Cronbach’s alpha	Dijkstra-Hensele’s *rho_A*	Composite reliability	Average variance extracted	Variance Inflation Factor
REL	4	0.962	0.964	0.973	0.899	1.695
ASE	4	0.964	0.967	0.974	0.903	1.529
SCT	4	0.963	0.964	0.973	0.900	1.534
MFQ	4	0.962	0.962	0.972	0.897	1.610
SOP	4	0.964	0.964	0.973	0.902	1.498
TRY	4	0.961	0.963	0.972	0.896	1.487
PSV	4	0.957	0.958	0.968	0.885	1.504
SAT	4	0.963	0.964	0.973	0.901	1.000
ITR	4	0.960	0.960	0.971	0.892	-

**Note:** REL: Reliability; ASE: Assurance; SCT: Security; MFQ: Maintaining Food Quality; SOP: System Operation; TRY: Traceability; PSV: Perceived Service Value; SAT: Satisfaction; ITR: Intention to Reuse.

**Table 4:** Discriminant validity.

	REL	ASE	SCT	MFQ	SOP	TRY	PSV	SAT	ITR
*Fornell-Larcker criterion*									
REL	0.948								
ASE	0.485	0.950							
SCT	0.477	0.435	0.949						
MFQ	0.505	0.422	0.435	0.947					
SOP	0.415	0.386	0.385	0.432	0.950				
TRY	0.401	0.404	0.401	0.413	0.422	0.947			
PSV	0.412	0.384	0.401	0.420	0.441	0.431	0.941		
SAT	0.391	0.363	0.347	0.389	0.428	0.387	0.405	0.949	
ITR	0.544	0.495	0.520	0.509	0.536	0.508	0.501	0.523	0.945
*Heterotrait-monotrait ratio (HTMT)*									
REL	-								
ASE	0.504	-							
SCT	0.496	0.451	-						
MFQ	0.525	0.438	0.452	-					
SOP	0.430	0.400	0.399	0.449	-				
TRY	0.417	0.418	0.416	0.429	0.438	-			
PSV	0.430	0.400	0.417	0.438	0.459	0.449	-		
SAT	0.406	0.376	0.360	0.404	0.444	0.401	0.421	-	
ITR	0.566	0.513	0.540	0.529	0.558	0.529	0.522	0.544	-

**Note:** REL: Reliability; ASE: Assurance; SCT: Security; MFQ: Maintaining Food Quality; SOP: System Operation; TRY: Traceability; PSV: Perceived Service Value; SAT: Satisfaction; ITR: Intention to Reuse.

### Hypothesis testing

After obtaining the effective reliabilities and validity results for the model, the measurement estimation performed. The adjusted R^2^ for the outcome variable SAT with the seven input variables (REL, ASE, SCT, MFQ, SOP, TRY and PSV) explained 30.3% of variation in SAT. The predictive relevance (Q^2^) for this part of model was 0.269 predicting a medium predictive relevance [40]. The adjusted R^2^ for the for the SAT on the ITR can described the 27.4% of variance in ITR. The predictive relevance of the part of model was 0.243, depicting a medium predictive relevance [40].

The proposed study hypotheses were tested with a bootstrapping procedure using a sample size 5000 [30]. Multicollinearity did not significantly distort PLS estimation as the VIF varied between 1.509 and 2.229, which was below the threshold value of 5 following Hair et al. [30]. Based on Table 5, REL demonstrated a positive and statistically significant impact on customer SAT (β = 0.098, *p* < 0.05. Likewise, ASE (β = 0.081, *p* < 0.05), MFQ (β = 0.097, *p* < 0.05), SOP (β = 0.183, *p* < 0.05), TRY (β = 0.117, *p* < 0.05), and PSV (β = 0.142, *p* < 0.05) reflected a positive and statistically significant effect on SAT. Furthermore, the confidence intervals of H_1_, H_2_, H_4_, H_5_, H_6_, and H_7_ did not include the value 0 between the 5% and 95% confidence intervals, supporting these hypotheses. Although perceived SCT was positively associated with SAT, the correlation was not statistically significant (β = 0.183, p < 0.05) and did not support H_3_. Lastly, customer SAT positively affected SAT (β = 0.523, *p* < 0.05) and supported H_8_ with confidence intervals that did not contain 0.

**Table 5:** Hypothesis testing.

Hypothesis		Beta	Mean	Confidence Intervals	t-value	Sig.	*R* ^*2*^	*Q2*	*f* ^*2*^	Decision
H_1_	REL → SAT	0.098	0.099	(0.035, 0.163)	2.543	0.006			0.008	Supported
H_2_	ASE → SAT	0.081	0.081	(0.018, 0.144)	2.116	0.017			0.006	Supported
H_3_	SCT → SAT	0.049	0.049	(-0.011, 0.109)	1.322	0.093			0.002	Rejected
H_4_	MFQ → SAT	0.097	0.097	(0.035, 0.161)	2.541	0.006	0.303	0.269	0.008	Supported
H_5_	SOP → SAT	0.183	0.183	(0.123, 0.241)	5.084	0.000			0.032	Supported
H_6_	TRY → SAT	0.117	0.117	(0.059, 0.176)	3.254	0.001			0.013	Supported
H_7_	PSV → SAT	0.142	0.142	(0.081, 0.200)	3.853	0.000			0.019	Supported
H_8_	SAT → ITR	0.523	0.524	(0.480, 0.566)	19.771	0.000	0.274	0.243	0.377	Supported

**Note:** REL: Reliability; ASE: Assurance; SCT: Security; MFQ: Maintaining Food Quality; SOP: System Operation; TRY: Traceability; PSV: Perceived Service Value; SAT: Satisfaction; ITR: Intention to Reuse.

### Mediation analysis

The relationship between the REL and ITR was mediated by the SAT tested in the H_M1_. The results (as presented in Table 6) revealed that satisfaction significantly mediated the relationship between reliability and intention to reuse food delivery services. It offers the support to accept the H_M1_. Next, H_M2_ was postulated to estimate the mediating effect of SAT between the ASE and ITR. The finding suggests that the relationship between the assurance and intention to reuse is significantly mediated by satisfaction; hence, this study accept the H_M2_. Subsequent, the mediation of SAT between the association of SCT and ITR was tested in H_M3_. The results indicated an insignificant mediating effect in the relationship of security and the intention to reuse the food delivery services by satisfaction. Therefore, this study reject H_M3_. H_M4_ evaluates the association between the MFQ and ITR mediated by the SAT. The analysis depicts a significant mediational effect of SAT between the association of MFQ and ITR. Hence offer support to admit the H_M4_.

**Table 6:** Mediation analysis.

Hypothesis		Beta	Confidence Intervals	t-value	Sig.	Decision
H_M1_	REL → SAT → ITR	0.051	(0.012, 0.093)	2.479	0.013	Supported
H_M2_	ASE → SAT → ITR	0.042	(0.003, 0.082)	2.082	0.037	Supported
H_M3_	SCT → SAT → ITR	0.025	(-0.012, 0.065)	1.297	0.195	Rejected
H_M4_	MFQ → SAT → ITR	0.051	(0.010, 0.091)	2.469	0.014	Supported
H_M5_	SOP → SAT → ITR	0.096	(0.056, 0.137)	4.726	0.000	Supported
H_M6_	TRY → SAT → ITR	0.061	(0.024, 0.100)	3.138	0.002	Supported
H_M7_	PSV → SAT → ITR	0.074	(0.036, 0.112)	3.785	0.000	Supported

**Note:** REL: Reliability; ASE: Assurance; SCT: Security; MFQ: Maintaining Food Quality; SOP: System Operation; TRY: Traceability; PSV: Perceived Service Value; SAT: Satisfaction; ITR: Intention to Reuse.

Following, the mediation of SAT between the relationship of SOP and ITR was tested in H_M5_. The results specified that a significant mediating effect of satisfaction exists between the relationship of system operation and the intention to reuse the food delivery services. Consequently, this study accept the H_M5_. H_M6_ appraises the association between the TRY and ITR mediated by the SAT. The analysis describes the significant mediational effect of SAT on the association of TRY and ITR. Hence offer support to admit the H_M6_. Lastly, H_M7_ investigates the mediational effect of SAT on the relationship between PSV and ITR. The findings reveal that a significant mediational effect of SAT exists between the perceived service value and intention to reuse food delivery services.

## Discussions

This study aimed to determine which food delivery services reflected high customer SAT and ITR. Notably, the conceptual research model was validated by scientifically evaluating the data gathered in China. The first study outcome denoted a strong and positive REL-SAT relationship (β = 0.098, p < 0.05). In ensuring service quality, REL in the online food delivery sector enables customers to select a dependable delivery platform to place orders, food delivered in good condition, and the professionalism of the delivery staff. A customer who receives a well-packaged item from a courteous and professional deliveryman would be more inclined to experience higher service approbation and SAT levels. This finding corresponded to multiple studies, where highly dependable services tend to elevate customers’ SAT [4, 15, 16] and ITR of food delivery applications.

Regarding the extent to which the protection of service rights by companies and deliverymen could improve users’ SAT with the current food delivery service, the second research outcome revealed a significant and positive ASE-SAT link (β = 0.081, p < 0.05). Consumers are willing to make additional payments for food delivery services if they believe the desired food items would reach them at a reasonable price and delivery service. As a measure of whether the delivery process is up to standard, a high level of ASE significantly minimises the probability of customer complaints and improves their SAT following past literature [4, 15]. The SCT-SAT path depicts an insignificant relationship highlighting that the online food delivery consumer still doubts the food delivery with the security concerns. The charging of higher prices and mis-use of user data remains the top concern about the online food delivery customers [20].

The fourth research outcome denoted a significant MFQ-SAT association. Essentially, MFQ significantly and positively impacted users’ SAT. Food colour and taste change with prolonged exposure post-preparation, specifically for products with high temperature and storage requirements, which inevitably affect users’ experience and SAT levels. Parallel to past works, low food quality during the delivery process would impact consumers’ PSV and induce their dissatisfaction with the food delivery service [4, 41].

The study finding affirmed the positive and significant SOP-SAT correlation in alignment with past works [42, 43]. An optimal SOP, the ability to select and purchase items and delivery options (time, location, and pick-up), prove critical in the online service platform. Notably, a seamless and digitalised SOP enables customers to promptly identify appropriate products and place orders in food delivery applications. Customer SAT and appeal could be maximised with such a feature, encouraging them to reuse the platform.

The next research outcome revealed that TRY’s food delivery service positively affected users’ SAT (β = 0.117, p < 0.05). Such TRY facilitates merchants, users, and platforms to share information and provide customers with optimal, reliable, and accurate information. Relevant food information could be tracked in real-time and offered to the user post-order placement for high food delivery safety, reasonable mealtime arrangements, and low customer anxiety while waiting for their food items towards improved SAT. The study findings corresponded to those of Alalwan [10] and Cheng et al. [4] in denoting the essentiality of TRY for high customer SAT.

The research outcome implied a strong and positive PSV-SAT correlation (β = 0.142, p < 0.05): the most significant latent of all the model constructs. Consumers are sensitised to emotion-related service components rather than just their rational counterparts. Hence, the users’ PSV of food delivery platforms significantly influences their SAT. In other words, high PSV induces high SAT. The PSV in earlier works, an essential indicator of customer SAT [6], paralleled the current research outcomes.

Based on the seventh study outcome, consumers’ SAT with online food delivery is a crucial factor impacting their ITR. This situation is evident in past studies involving multiple sectors [28, 44]. Generally, highly satisfied people with the outcomes of their previous behaviours and experiences are more likely to continue doing so. This finding paralleled Alalwan’s [10] study outcome involving the positive impact of customer SAT on their ITR mobile food ordering applications.

The empirical outcomes derived from this study could not affirm the role of SCT in predicting users’ SAT. Essentially, the SCT of Chinese network platforms has rapidly developed in recent years with the steady growth of information security technology and close supervision of government departments. Additionally, the overall system of current food delivery platforms further optimised SCT. Following past literature, SCT is no longer the critical factor impacting customer SAT when the SCT level is higher, or users perceive lower SCT risks [45]. Concurrently, SCT does not appear to be a motivational factor that improves SAT but a hygiene element that guarantees it for network applications based on the two-factor theory [46].

## Implications

### Theoretical implications

The current literature review highlighted limited research on food delivery service quality with complex procedures [10] (Chan & Gao, 2021). As a nation with the largest population and the world’s largest food delivery platform transaction volume, the Chinese perspective of food delivery service requires a holistic understanding. This study expanded the current body of knowledge with insights into the key determinants of optimal food delivery service implementation on local and global scales. For example, the integration of Parasuraman et al.’s [13] SERVQUAL model with the novel attributes of the food delivery industry in this study led to the incorporation of key online delivery service quality measures. These determinants are extending the SERVQUAL model (i.e., SCT, MFQ, SOP, TRY), and incorporating the PSV as additional factor that can guage the customer satisfaction in online food delivery. The model application and assessment in online food delivery platforms within a contemporary scenario are uncommon in the service business.

Additionally, the current work estimates the mediational effect of SAT among the seven factors of food delivery service quality and the intention to reuse the food delivery services. Online food delivery service realiability, assurance, maintainance of food quality, system operation, traceability and service value signifincatly mediated by the consumer satisfaction on the reuse intention of the online food delivery services. However, it is important to note that the relationship between security and reuse intention insignificantly mediated by the satisfaction. It highlights the issues of security about the food delivery as well as about the mis-handling of users data by the food delivery services. Effort must be made to effectibvely use the user data and safeguard the user identiy in all possible manner.

### Practical implications

On practical and empirical grounds, the current work provided a holistic understanding of the key determinants to be considered in developing and promoting the food delivery sector. As the most influential factor in predicting SAT, the role of PSV should be duly regarded and examined to internalise customers’ overall emotional assessments of food delivery services. Marketers should allow users to feel the hospitality of food delivery services through detailed services, such as discounts or free orders for users from different age groups on special days and a sense of empathy. Such provisions could increase users’ sense of belonging to the platform. REL should be equally emphasised regarding food quality during the delivery process, from order placement to food delivery. The deliveryman’s professionalism in offering service would affect consumers’ perception of REL involving food delivery service quality. Thus, such individuals should be provided with relevant pre-job training to communicate with users from different backgrounds and regularly inspect delivery vehicles, deliverymen, and delivery boxes to ensure food hygiene during delivery.

The ASE factor should also be seriously regarded. For example, digital food delivery platforms could establish specific insurance mechanisms, which could be customised for users from different education levels, thus inducing higher ASE. Consumers who encounter (i) order timeout and product loss post-order placement and (ii) food quality issues can apply for compensation on the platform. Preferential and convenient services could also be offered to such users. These platforms should also gather feedback from these customers for high consumer SAT. In ensuring the integrity of the final delivery and food appearance to improve user SAT, MFQ is another factor that concerns users. A delivery process that results in spilling, dripping, squeezing, cooling and other mishaps could leave a bad first impression on the service recipients and induce low SAT and ITR in addition to the implications of the food taste caused by prolonged delivery time. Firstly, the distribution box’s capacity, vibration resistance, and insulation should be improved to avoid compromising the food integrity owing to adverse factors in the distribution process. Second, the platform could collaborate with merchants to provide various packaging boxes for different meal and beverage types and fulfil the multiple food package requirements. Therefore, consumers can try to maintain the same taste when they enjoy takeout as they do in a restaurant.

A seamless SOP enables customers to complete orders and successfully elevate their ITR through efficiency. Thus, food delivery platforms should simplify ordering, increase the matching degree between pictures and dish names, provide consumers with the most appealing visual experience, and optimise platform ordering systems to cater to users from different education levels. Meanwhile, TRY requires customers to track the entire order status and real-time delivery using relevant platforms. Although a significant number of existing platforms support food delivery status tracking, the food preparation process and time duration consumed by the merchants are not digitally available. The amount of meal preparation time consumed by merchants is also one of the reasons underpinning delayed food delivery despite accurate estimates and monitoring. Hence, marketers should incorporate the meal preparation time and process into the scope of online food delivery TRY and update merchants’ meal delivery progress in real-time. The ordered food items should also be visually displayed and uploaded to the system post-order completion to prevent mismatches between the order and the actual food delivered.

## Conclusions

While prior research has primarily concentrated on assessing customer satisfaction with food delivery platforms and exploring the impact of certain platform-related factors on reuse behavior, this study takes a comprehensive approach by examining the various factors that influence customers’ satisfaction and intention to reuse food delivery services, specifically focusing on the quality of the service. Additionally, this study also investigates the mediating role of SAT in the relationship between these factors and ITR. The findings indicate a significant relationship between the majority of factors related to the quality of delivery service and the levels of consumer satisfaction and intention to repurchase. The comprehensive examination of REL, ASE, MFQ, SOP, TRY, and PSV has demonstrated that these factors play a crucial role in enhancing user satisfaction with food delivery services. Except for the association between SCT and ITR, SAT serves as a positive mediator for the impact of other factors on ITR. Theoretically, consider that individual perception factors play an important role in how consumers make decisions, this study introduces PSV as an extension factor of the SERVQUAL model based on the characteristics of the food delivery industry and proves that it has a positive effect on SAT and ITR. This finding offers a novel avenue of inquiry for future researchers to investigate the psychological factors related to the utilization of food delivery services. Practically, as a pioneer in the global food delivery industry, China acts as a benchmark for market growth to a certain extent. A better understanding of the factors influencing consumer behavior in the Chinese food delivery industry can not only contribute to the improvement of the overall service level of the industry but also serve as a guide for the strategic market decisions of enterprises in other countries.

Despite providing pivotal insights to understand the factors impacting Chinese consumers’ SAT and ITR involving online food delivery services, this study encountered several limitations. First, the current work was performed cross-sectionally, with primary data gathered within a specified timeframe, thus making it impossible to depict users’ attitudes and perspectives, which may change over time. Future scholars could perform longitudinal and qualitative research to explore how consumers’ SAT and ITR gradually shift. Although a sizable number of factors have been incorporated into this research, further research may consider other factors involving product diversity, personalisation and culture that may influence the satisfaction and reuse intention. The consumer level factors of economic, social psychological also helps in the formation of satisfaction and reuse intention that are necessary to study in future study. The current study findings, which only involved one country (China) in preventing potential cross-cultural bias, could be empirically compared against nations with similar characteristics for outcome optimisation and a broad spectrum of practice decisions.

## Supporting information

S1 Table Survey instrument. (DOCX)

S2 Table Loading and cross loadings. (DOCX)

S3 Table Dataset. (CSV)

## References

[1] Verma P. (2020). The effect of presentation, product availability and ease upon transaction reliability for online food delivery aggregator applications–moderated mediated model. *Journal of Foodservice Business Research*, 23(4), 285–304. 10.1080/15378020.2020.1761586

[2] Suresh S., & Vasantha S. (2020). Influence of logistics service quality among customer satisfaction using IOT based techniques. *Materials Today*: *Proceedings*. 10.1016/j.matpr.2020.11.764

[3] Shankar A., Jebarajakirthy C., Nayal P., Maseeh H. I., Kumar A., & Sivapalan A. (2022). Online food delivery: A systematic synthesis of literature and a framework development. *International Journal of Hospitality Management*, 104, 103240. 10.1016/j.ijhm.2022.103240

[4] Cheng C.-C., Chang Y.-Y., & Chen C.-T. (2021). Construction of a service quality scale for the online food delivery industry. *International Journal of Hospitality Management*, 95. 10.1016/j.ijhm.2021.102938

[5] Chan J., & Gao Y. L. (2021). Measuring the up-to-date quality of online food delivery: Formative index construction. *International Journal of Contemporary Hospitality Management*, 33(12), 4550–4568. 10.1108/IJCHM-06-2021-0739

[6] Aslam W., Ham M., & Arif I. (2021). Technology at the dining table: Linking perceived value, service recovery, and continuous intention to use food delivery applications. *Review of Business Management*, 23(4), 600–618. 10.7819/rbgn.v23i4.4135

[7] Erkmen E., & Turegun N. (2022). Success model of online food delivery system: The role of brand image in customer responses. In *Innovative Marketing* (Vol. 18, Issue 2, pp. 148–160). LLC CPC Business Perspectives. 10.21511/im.18(2).2022.13

[8] China Internet Network Information Center. (2023). *The 51st Statistical Report on China’s Internet Development*. China Internet Network Information Center(CNNIC). Retrieved June 9, 2023, from https://www.cnnic.net.cn/n4/2023/0303/c88-10757.html.

[9] Annaraud K., & Berezina K. (2020). Predicting satisfaction and intentions to use online food delivery: What really makes a difference? *Journal of Foodservice Business Research*, 23(4), 305–323. 10.1080/15378020.2020.1768039

[10] Alalwan A. A. (2020). Mobile food ordering apps: An empirical study of the factors affecting customer e-satisfaction and continued intention to reuse. *International Journal of Information Management*, 50, 28–44. 10.1016/j.ijinfomgt.2019.04.008

[11] Choi Y., Zhang L., Debbarma J., & Lee H. (2021). Sustainable management of online to offline delivery apps for consumers’ reuse intention: Focused on the meituan apps. *Sustainability (Switzerland)*, 13(7). Scopus. 10.3390/su13073593

[12] Febrianti R. A. M., Khan A. R., Alde R., Novanka R., Therawan A., & Firmanto A. (2021). Understanding Intention to Use Online Delivery Food in Go Food Application. In *Review of International Geographical Education Online* (Vol. 11, Issue 6, pp. 791–798). Eskisehir Osmangazi University. 10.48047/rigeo.11.06.97

[13] Parasuraman A., Zeithaml V. A., & Berry L. L. (1985). A Conceptual Model of Service Quality and Its Implications for Future Research. *Journal of Marketing*, 49(4), 41–50. 10.2307/1251430

[14] German J. D., Redi A. A. N. P., Prasetyo Y. T., Persada S. F., Ong A. K. S., Young M. N., et al. (2022). Choosing a package carrier during COVID-19 pandemic: An integration of pro-environmental planned behavior (PEPB) theory and service quality (SERVQUAL). *Journal of Cleaner Production*, 346, 131123. doi: 10.1016/j.jclepro.2022.131123 35281884 PMC8898924

[15] Kian Y.K., Cheah H., & Chang Y. (2022). A model of online food delivery service quality, customer satisfaction, and customer loyalty: A combination of PLS-SEM and NCA approaches. *British Food Journal*. 10.1108/BFJ-10-2021-1169

[16] Boshoff C. (2007). A Psychometric Assessment of E-S-Qual: A Scale to Measure Electronic Service Quality. *Journal of Electronic Commerce Research*, 8(1), 101–114.

[17] Parasuraman A., Zeithaml V. a., & Malhotra A. (2005). E-S-QUAL a multiple-item scale for assessing electronic service quality. *Journal of Service Research*, 7(3), 213–233. 10.1177/1094670504271156

[18] Yang Q., Al Mamun A., Hayat N., Salleh M. F., M. F. Md., Jingzu, G., & Zainol N. R (2022). Modelling the mass adoption potential of wearable medical devices. *PLoS ONE*, 17(6), 1–18. doi: 10.1371/journal.pone.0269256 35675373 PMC9176812

[19] Strzelecki A. & Rizun M. (2020). Consumers’ security and trust for online shopping after GDPR: Examples from Poland and Ukraine. *Digital Policy*, *Regulation and Governance*, 22(4), 289–305. 10.1108/DPRG-06-2019-0044

[20] Sharma S. K., Sharma H., & Dwivedi Y. K. (2019). A Hybrid SEM-Neural Network Model for Predicting Determinants of Mobile Payment Services. *Information Systems Management*, 36(3), 243–261. 10.1080/10580530.2019.1620504

[21] McCole P., Ramsey E., & Williams J. (2010). Trust considerations on attitudes towards online purchasing: The moderating effect of privacy and security concerns. *Journal of Business Research*, 63(9), 1018–1024. 10.1016/j.jbusres.2009.02.025

[22] Al Amin M., Arefin M. S., Alam M. R., Ahammad T., & Hoque M. R. (2021). Using Mobile Food Delivery Applications during COVID-19 Pandemic: An Extended Model of Planned Behavior. *Journal of Food Products Marketing*, 27(2), 105–126. 10.1080/10454446.2021.1906817

[23] Wen H., Sanjukta P., & Bharath M. J. (2021). A comprehensive examination of consumers’ intentions to use food delivery apps. *British Food Journal*, 124(5), 1737–1754. 10.1108/BFJ-06-2021-0655

[24] Pal D., Funilkul S., Eamsinvattana W., & Siyal S. (2021). Using online food delivery applications during the COVID-19 lockdown period: What drives University Students’ satisfaction and loyalty? *Journal of Foodservice Business Research*, 1–45. 10.1080/15378020.2021.1964419

[25] Jin S., & Zhou L. (2014). Consumer interest in information provided by food traceability systems in Japan. *Food Quality and Preference*, 36, 144–152. 10.1016/j.foodqual.2014.04.005

[26] Zeithaml V. (1988). Consumer Perceptions of Price, Quality, and Value—A Means-End Model and Synthesis of Evidence. *Journal of Marketing*, 52(3), 2–22. 10.2307/1251446

[27] Bonsón-Ponte E., Carvajal-Trujillo E., & Escobar-Rodríguez T. (2015). Influence of trust and perceived value on the intention to purchase travel online: Integrating the effects of assurance on trust antecedents. *Tourism Management*, 47, 286–302. 10.1016/j.tourman.2014.10.009

[28] Lee S.-H., Kwak M.-K., & Cha S.-S. (2020). Consumers’ choice for fresh food at online shopping in the time of covid19. In *Journal of Distribution Science* (Vol. 18, Issue 9, pp. 45–53). Korea Distribution Science Association (KODISA). 10.15722/jds.18.9.202009.45

[29] Oliver R. L. (1980). A Cognitive Model of the Antecedents and Consequences of Satisfaction Decisions. *Journal of Marketing Research (JMR)*, 17(4), 460–469. 10.2307/3150499

[30] Hair J.F, Sarstedt M., Ringle C., M. & Gudergan S., P. (2017). *Advanced Issues in Partial Least Squares Structural Equation Modeling*. SAGE Publications, Inc.

[31] Kumar A., Adlakaha A., & Mukherjee K. (2018). The effect of perceived security and grievance redressal on continuance intention to use M-wallets in a developing country. *International Journal of Bank Marketing*, 36(7), 1170–1189. 10.1108/ijbm-04-2017-0077

[32] Kim S., Bae J., & Jeon H. (2019). Continuous intention on accommodation apps: Integrated Value-based adoption and expectation–confirmation model analysis. *Sustainability*, 11(6), 1578. 10.3390/su11061578

[33] Cappelli L., Guglielmetti R., Mattia G., Merli R., & Francesca Renzi M. (2011). Testing a customer satisfaction model for online services. *International Journal of Quality and Service Sciences*, 3(1), 69–92. 10.1108/17566691111115090

[34] Kang J.-W., & Namkung Y. (2019). The information quality and source credibility matter in customers’ evaluation toward Food O2O Commerce. *International Journal of Hospitality Management*, 78, 189–198. 10.1016/j.ijhm.2018.10.011

[35] Rodríguez-Ardura I., & Meseguer-Artola A. (2020). Editorial: How to Prevent, Detect and Control Common Method Variance in Electronic Commerce Research. *Journal of Theoretical & Applied Electronic Commerce Research*, 15(2), i–v. 10.4067/S0718-18762020000200101

[36] Kock N. (2015). Common method bias in PLS-SEM: A full collinearity assessment approach. *International Journal of E-Collaboration*, 11, 1–10. 10.4018/ijec.2015100101

[37] Memon M. A., Cheah J.-H., Ramayah T., Ting H., & Chuah F. (2018). Mediation analysis: Issues and recommendations. *Journal of Applied Structural Equation Modeling*, 2(1), i–ix. 10.47263/jasem.2(1)01

[38] Hair J. F., Astrachan C. B., Moisescu O. I., Radomir L., Sarstedt M., Vaithilingam S., et al. (2021). Executing and interpreting applications of PLS-SEM: Updates for family business researchers. *Journal of Family Business Strategy*, 12(3), 100392. 10.1016/j.jfbs.2020.100392

[39] Fornell C., & Larcker D. F. (1981). Evaluating structural equation models with unobservable variables and measurement error. *Journal of Marketing Research*, 18(1), 39. 10.2307/3151312

[40] Chin W. W. (2009). How to write up and report PLS analyses. *Handbook of Partial Least Squares*, 655–690. 10.1007/978-3-540-32827-8_29

[41] Dospinescu N., Dospinescu O., & Tatarusanu M. (2020). Analysis of the Influence Factors on the Reputation of Food-Delivery Companies: Evidence from Romania. *Sustainability (2071–1050)*, 12(10), 4142. 10.3390/su12104142

[42] Ahn J. (2021). Exploring perceived innovation in building customers’ patronizing behavior in the food delivery service context. *International Journal of Quality and Service Sciences*, 14(2), 258–273. 10.1108/IJQSS-08-2021-0114

[43] Xue G., Wang Z., & Wang G. (2021). Optimization of Rider Scheduling for a Food Delivery Service in O2O Business. *Journal of Advanced Transportation*, 1–15. 10.1155/2021/5515909

[44] Wang Y.-S., Tseng T. H., Wang W.-T., Shih Y.-W., & Chan P.-Y. (2019). Developing and validating a mobile catering app success model. *International Journal of Hospitality Management*, 77, 19–30. 10.1016/j.ijhm.2018.06.002

[45] Hanif Y., & Lallie H. S. (2021). Security factors on the intention to use mobile banking applications in the UK older generation (55+). A mixed-method study using modified UTAUT and MTAM—with perceived cyber security, risk, and trust. *Technology in Society*, 67. 10.1016/j.techsoc.2021.101693

[46] Lo L. Y.-S., Lin S.-W., & Hsu L.-Y. (2016). Motivation for online impulse buying: A two-factor theory perspective. *International Journal of Information Management*, 36(5), 759–772. 10.1016/j.ijinfomgt.2016.04.012

