# Supplementary material for: Modelling the significance of food delivery service quality on customer satisfaction and reuse intention
Source: PLoS One. 2024 Feb 15;19(2):e0293914. doi: 10.1371/journal.pone.0293914 (PMC10868767; doi:10.1371/journal.pone.0293914)
Supplement: S1 Table — (DOCX) [file pone.0293914.s001.docx]

**S1 Table.** Survey Instrument

| REL1 | The delivery box is in good condition. | |
| --- | --- | --- |
| REL2 | The attire of the deliveryman is neat and clean. | |
| REL3 | The deliveryman’s words and behaviors is encouraging. | |
| REL4 | The bills or details were supplied correctly by the deliveryman. | |
| ASE1 | The food is always delivered on time. | |
| ASE2 | The food was always delivered in the correct quantity. | |
| ASE3 | The deliverymen are well trained to deliver the food correctly. | |
| ASE4 | The deliveryman is well trained and experienced. | |
| SCT1 | The food delivery application protects customers’ personal information | |
| SCT2 | The food delivery application protects customers’ credit card and/or electronic payment information | |
| SCT3 | The food delivery application keeps customers’ ordering records private | |
| SCT4 | Buying food with food delivery application is safe. | |
| MFQ1 | At the time of delivery, the food smells delicious. | |
| MFQ2 | The food temperature remains good at the time of delivery. | |
| MFQ3 | The food appearance was maintained at the time of delivery. | |
| MFQ4 | The flavor of the food is consistent at the time of delivery. | |
| SOP1 | The food delivery application offers appropriate instructions. | |
| SOP2 | The use of food delivery applications is simple and straight-forward. | |
| SOP3 | The food delivery application helps find the meal. | |
| SOP4 | The food delivery application offers suitable meal options to the customers. | |
| TRY1 | Apps for food delivery provide delivery information. | |
| TRY2 | The expected time of food delivery is provided. | |
| TRY3 | The deliveryman quickly responds to the food delivery order. | |
| TRY4 | I can easily track the delivery of the food. | |
| PSV1 | Food delivery services are reasonably priced. | |
| PSV2 | Food delivery services are beneficial | |
| PSV3 | Food delivery services help me to be more efficient | |
| PSV4 | Food delivery services help me save time | |
| SAT1 | I I can easily use the food delivery application . | |
| SAT2 | I found all the information on food delivery without having to go anywhere else. | |
| SAT3 | Using the food delivery application is more advantageous than traditional channels. | |
| SAT4 | Food delivery application has simplified my activities. | |
| ITR1 | | I would choose the food delivery application as a preference to purchase food products. |
| ITR2 | | I would be willing to use the food delivery application to purchase food products. |
| ITR3 | | I would recommend the food delivery application to others. |
| ITR4 | | I plan to use the food delivery application in the future. |
