# Supplementary material for: Modelling the significance of food delivery service quality on customer satisfaction and reuse intention
Source: PLoS One. 2024 Feb 15;19(2):e0293914. doi: 10.1371/journal.pone.0293914 (PMC10868767; doi:10.1371/journal.pone.0293914)
Supplement: S2 Table — (DOCX) [file pone.0293914.s002.docx]

**S2 Table.** Loading and Cross Loadings

|  | REL | ASE | SCT | MFQ | SOP | TRY | PSV | SAT | ITR |
| --- | --- | --- | --- | --- | --- | --- | --- | --- | --- |
| REL1 | 0.976 | 0.470 | 0.454 | 0.483 | 0.400 | 0.391 | 0.401 | 0.388 | 0.535 |
| REL2 | 0.942 | 0.472 | 0.451 | 0.486 | 0.393 | 0.386 | 0.405 | 0.367 | 0.513 |
| REL3 | 0.934 | 0.449 | 0.438 | 0.472 | 0.381 | 0.373 | 0.370 | 0.350 | 0.488 |
| REL4 | 0.940 | 0.449 | 0.466 | 0.475 | 0.397 | 0.370 | 0.386 | 0.376 | 0.526 |
| ASE1 | 0.469 | 0.978 | 0.414 | 0.415 | 0.384 | 0.407 | 0.387 | 0.362 | 0.495 |
| ASE2 | 0.454 | 0.942 | 0.417 | 0.397 | 0.359 | 0.378 | 0.359 | 0.335 | 0.464 |
| ASE3 | 0.469 | 0.946 | 0.417 | 0.414 | 0.378 | 0.399 | 0.379 | 0.359 | 0.473 |
| ASE4 | 0.452 | 0.936 | 0.405 | 0.375 | 0.345 | 0.347 | 0.333 | 0.320 | 0.447 |
| SCT1 | 0.471 | 0.434 | 0.978 | 0.429 | 0.387 | 0.403 | 0.403 | 0.346 | 0.524 |
| SCT2 | 0.457 | 0.403 | 0.941 | 0.415 | 0.355 | 0.376 | 0.365 | 0.324 | 0.485 |
| SCT3 | 0.426 | 0.395 | 0.941 | 0.401 | 0.345 | 0.366 | 0.361 | 0.317 | 0.475 |
| SCT4 | 0.456 | 0.417 | 0.936 | 0.404 | 0.374 | 0.374 | 0.390 | 0.329 | 0.489 |
| MFQ1 | 0.510 | 0.422 | 0.434 | 0.975 | 0.427 | 0.405 | 0.407 | 0.379 | 0.502 |
| MFQ2 | 0.466 | 0.398 | 0.411 | 0.941 | 0.398 | 0.377 | 0.393 | 0.371 | 0.483 |
| MFQ3 | 0.486 | 0.393 | 0.408 | 0.940 | 0.417 | 0.385 | 0.398 | 0.361 | 0.478 |
| MFQ4 | 0.452 | 0.385 | 0.393 | 0.932 | 0.395 | 0.397 | 0.396 | 0.364 | 0.464 |
| SOP1 | 0.416 | 0.389 | 0.390 | 0.433 | 0.978 | 0.409 | 0.429 | 0.414 | 0.524 |
| SOP2 | 0.388 | 0.366 | 0.362 | 0.399 | 0.942 | 0.415 | 0.428 | 0.407 | 0.491 |
| SOP3 | 0.387 | 0.364 | 0.361 | 0.396 | 0.941 | 0.399 | 0.401 | 0.409 | 0.504 |
| SOP4 | 0.383 | 0.348 | 0.350 | 0.412 | 0.937 | 0.378 | 0.416 | 0.393 | 0.517 |
| TRY1 | 0.408 | 0.413 | 0.410 | 0.416 | 0.414 | 0.975 | 0.427 | 0.369 | 0.499 |
| TRY2 | 0.367 | 0.389 | 0.381 | 0.388 | 0.400 | 0.940 | 0.406 | 0.374 | 0.475 |
| TRY3 | 0.363 | 0.361 | 0.363 | 0.395 | 0.389 | 0.940 | 0.404 | 0.379 | 0.480 |
| TRY4 | 0.380 | 0.365 | 0.362 | 0.363 | 0.393 | 0.930 | 0.396 | 0.341 | 0.468 |
| PSV1 | 0.431 | 0.390 | 0.415 | 0.418 | 0.427 | 0.415 | 0.969 | 0.377 | 0.486 |
| PSV2 | 0.384 | 0.368 | 0.385 | 0.401 | 0.413 | 0.403 | 0.934 | 0.378 | 0.466 |
| PSV3 | 0.363 | 0.351 | 0.352 | 0.377 | 0.389 | 0.382 | 0.926 | 0.365 | 0.460 |
| PSV4 | 0.373 | 0.338 | 0.356 | 0.386 | 0.427 | 0.420 | 0.933 | 0.401 | 0.470 |
| SAT1 | 0.387 | 0.367 | 0.346 | 0.382 | 0.416 | 0.380 | 0.394 | 0.981 | 0.511 |
| SAT2 | 0.372 | 0.331 | 0.325 | 0.360 | 0.392 | 0.359 | 0.369 | 0.939 | 0.487 |
| SAT3 | 0.364 | 0.342 | 0.319 | 0.359 | 0.398 | 0.363 | 0.378 | 0.940 | 0.498 |
| SAT4 | 0.362 | 0.337 | 0.327 | 0.376 | 0.417 | 0.367 | 0.395 | 0.936 | 0.490 |
| ITR1 | 0.551 | 0.502 | 0.529 | 0.515 | 0.536 | 0.503 | 0.503 | 0.512 | 0.977 |
| ITR2 | 0.502 | 0.447 | 0.480 | 0.490 | 0.483 | 0.468 | 0.450 | 0.492 | 0.934 |
| ITR3 | 0.510 | 0.472 | 0.493 | 0.459 | 0.495 | 0.479 | 0.475 | 0.494 | 0.934 |
| ITR4 | 0.491 | 0.447 | 0.462 | 0.456 | 0.510 | 0.469 | 0.463 | 0.478 | 0.933 |

**Note:** REL: Reliability; ASE: Assurance; SCT: Security; MFQ: Maintaining Food Quality; SOP: System Operation; TRY: Traceability; PSV: Perceived Service Value; SAT: Satisfaction; ITR: Intention to Reuse.
